# Iliopsoas Tendon Reformation after Psoas Tendon Release

**DOI:** 10.1155/2013/361087

**Published:** 2013-04-15

**Authors:** K. Garala, R. A. Power

**Affiliations:** University Hospitals Leicester, Leicester General Hospital, Gwendolen Road, Leicester LE5 4PW, UK

## Abstract

Internal snapping hip syndrome, or psoas tendonitis, is a recognised cause of nonarthritic hip pain. The majority of patients are treated conservatively; however, occasionally patients require surgical intervention. The two surgical options for iliopsoas tendinopathy are step lengthening of the iliopsoas tendon or releasing the tendon at the lesser trochanter. Although unusual, refractory snapping usually occurs soon after tenotomy. We report a case of a 47-year-old active female with internal snapping and pain following an open psoas tenotomy. Postoperatively she was symptom free for 13 years. An MRI arthrogram revealed reformation of a pseudo iliopsoas tendon reinserting into the lesser trochanter. The pain and snapping resolved after repeat iliopsoas tendon release. Reformation of tendons is an uncommon sequela of tenotomies. However the lack of long-term studies makes it difficult to calculate prevalence rates. Tendon reformation should be included in the differential diagnosis of failed tenotomy procedures after a period of symptom relief.

## 1. Introduction

Iliopsoas tendinitis is the inflammation and thickening of the iliopsoas tendon sheath over the iliopectineal ridge. In some patients the tendon sheath causes a snapping sensation when it flicks over the iliopectineal bar or the iliacus tendon. It is encompassed within the spectrum of snapping hip syndrome [1]. Iliopsoas tendinitis can be a cause of the less common, internal snapping hip syndrome [2]. Primary iliopsoas tendinitis is a disease of overuse; however it does occur secondary to hip arthroplasty surgery [3, 4]. It is usually managed conservatively by rest, physiotherapy, analgesic, and NSAIDs. If conservative management fails, then a fluoroscopically guided injection of steroids and local anaesthetic into the hip are offered and surgery is considered.

Two procedures have been described previously in the literature. One operation involves lengthening the psoas muscle tendon accomplished by the step cutting of the tendinous portion of the iliopsoas [5]. The second option is surgically releasing the iliopsoas tendon by dividing it from its attachment to the lesser trochanter [6]. This can be done openly or endoscopically [7]. Both of these operations aim to relieve friction on the tendon when it rubs on the iliopectineal bar.

In this paper we report a patient who required a second psoas release due to reformation of the iliopsoas tendon.

## 2. Case Description

A 47-year-old active lady presented with a history of pain, stiffness, and instability in her left hip. She also complained of difficulty in wearing shoes and other high flexion movements. She had no symptoms in her right hip. 13 years prior to this presentation she was diagnosed with bilateral iliopsoas tendinopathy. After confirmation with tenography and MRI arthrogram, she underwent psoas release procedures on both hips.

On examination there was no obvious swelling in the groin. There was no leg length discrepancy and no fixed flexion deformity. The patient had an uncomfortable straight leg raise. She had significant impingement pain on FABER test but had no obvious snapping suggesting irritability of the psoas tendon.

**Figure 1:**
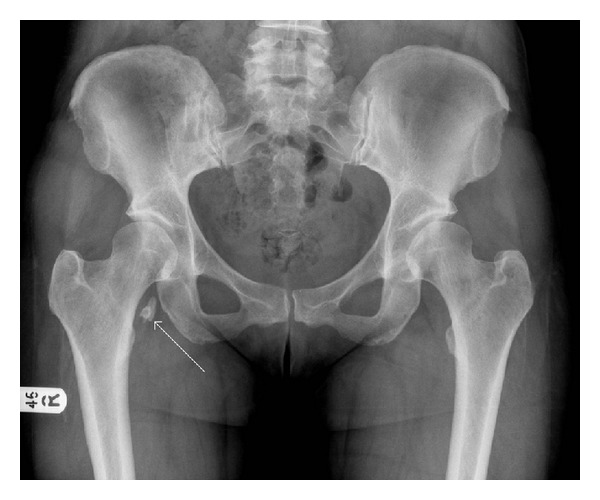
An anterior-posterior plain radiograph of the patient's hips taken 3 months prior to the repeat left psoas release surgery. There are only mild dysplastic changes present in the left hip. There is significant ossification, demonstrated by the arrow, superior to the right lesser trochanter, which is likely secondary to the patient's right psoas tenotomy undertaken 11 years ago.

**Figure 2:**
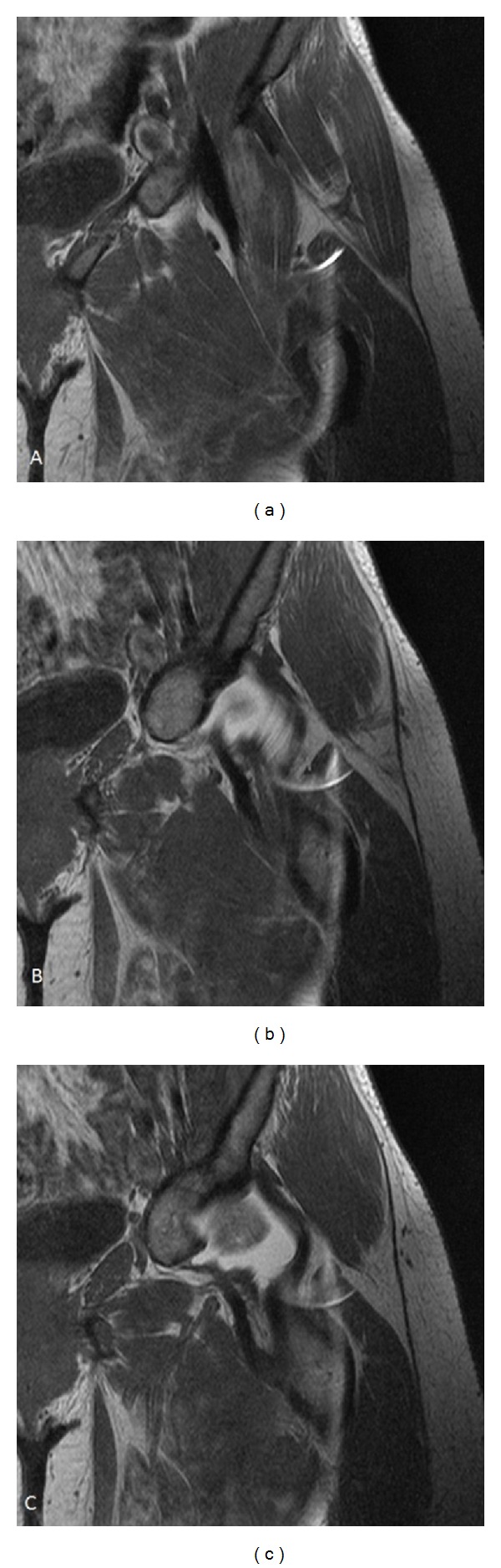
A series of T2-weighted, coronal MR arthrograms of the left hip taken 3 months prior to the patient's repeat left psoas release. The images identify an iliopsoas pseudo-tendon anterior to the hip joint (a). This pseudo-tendon extends beyond the hip joint (b) and is inserted into the superior aspect of the lesser trochanter (c).

Radiographs (Figure 1) showed mild dysplastic changes in her left hip and ossification of her severed psoas tendon in the right hip. An MRI scan (Figure 2) of her left hip revealed a pseudo iliopsoas tendon inserted into the lesser trochanter. After a period of failed conservative management, fluoroscopically guided injections with steroid and anaesthetic provided pain relief for a short period of time. Following this, the patient underwent a repeat left psoas release.

A 4 cm incision was made in the groin crease medial to the proximal insertion of the adductor longus tendon. After identifying the lesser trochanter, blunt dissection was used to remove surrounding soft tissue thus isolating the iliopsoas pseudo-tendon. The tendon was then cut with scissors and finally the wound was closed.

After this procedure the patient reported that pain and stiffness had improved. After 6 months, she was able to walk and jog with minimal discomfort in her hip.

## 3. Discussion

There have been no previous reports of iliopsoas tendon reformation after psoas release surgery in the literature. Spontaneous tendon reformation is uncommon. The normal outcome of psoas releases is that the tendon remains severed ensuring decreased traction upon the original bone to which it was attached. There is one study on adductor tenotomies revealing 3 out of 109 patients who underwent surgery had their adductor tendons reform after surgery [8].

Tendon rupture and repair are heavily studied due to the prevalence of Achilles tendon ruptures. There has been much research into methods to induce Achilles tendon repair [9, 10]. This is a difficult injury to treat. Surgical repair of the tendon is preferable to conservative management due to lower rerupture rates (3.1% versus 13%, resp.) [11]. Platelet rich plasma has been considered as a tool to improve Achilles tendon reconstruction. Activated platelets release a wide array of growth factors which could initiate and enhance repair pathways. Platelet concentrate injection improved Achilles tendon repair in rats [12]. There was increased strength and stiffness in tendons treated with platelets compared with those not treated with platelet concentrate. This translates well in humans. Patients treated with platelet rich fibre matrices placed on their sutured Achilles tendon recovered faster than patients with simple sutured tendons [13]. These studies suggest that platelets augment the healing process of tendons. Our patient developed a haematoma around the stub of her left iliopsoas tendon at the initial tenotomy. This may have caused sufficient release of platelet derived growth factors aiding tendon reformation after psoas tenotomy.

Another interesting aspect of this case is the response of the right psoas tendon to psoas tenotomy. 10 years after her psoas release surgery, she had few complaints with her right hip. The plain radiograph of her hips revealed significant ossification superior-medial to her right lesser trochanter. Ossification of ruptured Achilles tendons has been reported before [14]. With regard to psoas tendons, one study reports that 23.2% of hips treated by arthroplasty and psoas tenotomy had heterotopic ossification [15]. Hips treated with solely arthroplasty had no cases of ossification. The authors state the most likely cause of this is the manner by which the iliopsoas tendon is elevated from the lesser trochanter by stripping, as opposed to sharp section with diathermy, scissors, or scalpel [15]. This technique causes periosteal stripping which may lead to the heterotopic ossification. The iliopsoas tendon in this patient was separated with scissors. There would therefore be minimal periosteal stripping; thus this does not explain the extent of ossification which occurred in this patient's right psoas tendon. There is also a reported case of ossification following a psoas lengthening procedure [16].

There are few studies investigating the long-term effects of psoas release surgery. A 22-patient study of surgical release of snapping iliopsoas has a mean followup of 17 months [6]. Weakness is the only complication mentioned in this study. There are small studies which examine the effectiveness of arthroscopic psoas tendon releases; however these are only medium term at best [17–20]. The complications of our patients began 13 years after her original operation. There is one case report of a patient with snapping after psoas tendon release [21]. The cause of this patient's refractory internal snapping was a bifid iliopsoas tendon. Complications are also noted in psoas lengthening procedures. In one 20-year study investigating the long-term results of psoas lengthening, 40 complications were reported from a total of 92 cases [22]. Some cases required further surgical intervention. This technique cannot be compared to the psoas release surgery our patient underwent because psoas lengthening preserves the attachment of the iliopsoas tendon to the lesser trochanter. There is a need for further research studying the long-term response of patients to psoas release surgery.

We conclude that this is the first reported case of iliopsoas tendon reformation. It is important for surgeons to consider the possibility of tendon reformation in patients who have undergone tenotomies previously.
